# Milk protein IgG and IgA: the association with milk-induced gastrointestinal symptoms in adults

**DOI:** 10.1186/2045-7022-1-S1-P39

**Published:** 2011-08-12

**Authors:** Marina Petroviæ

**Affiliations:** 1 Clinical Center Kragujevac, Pulmonary, Kragujevac, Serbia

## Backround

To study the association between serum levels of milk protein IgG and IgA antibodies and milk-related gastrointestinal symptoms in adults.

## Methods

Milk protein IgG and IgA antibodies were determined in serum samples of 270 subjects. Subjects were randomly selected from a total of 1560 adults undergoing laboratory investigations in primary care. All 270 participants had completed a questionnaire on abdominal symptoms and dairy consumption while waiting for the laboratory visit. The questionnaire covered the nature and frequency of gastrointestinal problems, the provoking food items, family history and allergies. The levels of specific milk protein IgG and IgA were measured by using the ELISA technique. The association of the milk protein-specific antibody level was studied in relation to the milk-related gastrointestinal symptoms and dairy consumption.

## Results

Subjects drinking milk (n= 45) had higher levels of milk protein IgG in their sera than non-milk drinkers (n=125, P<0.001). Subjects with gastrointestinal problems related to milk drinking consumed less milk but had higher milk protein IgG levels than those with no milk-related gastrointestinal symptoms (P=0.03). Among the symptomatic subjects, those reporting dyspeptic symptoms had lower milk protein IgG levels than non-dyspeptics (P<0.05). However, dyspepsia was not associated with milk drinking (P=0.59). The association of high milk protein IgG levels with constipation was close to the level of statistical significance. Diarrhea had no association with milk protein IgG level (P=0.5). With regard to minor symptoms, flatulence and bloating (P=0.92), were not associated with milk protein IgG level. Milk protein IgA levels did not show any association with milk drinking or abdominal symptoms. The levels of milk protein IgA and IgG declined as the age of the subjects increased (P<0.001).

## Conclusion

Milk protein IgG but not milk IgA seems to be associated with self-reported milk-induced gastrointestinal symptoms.

